# Disturbances in Muscle Energy Metabolism in Patients with Amyotrophic Lateral Sclerosis

**DOI:** 10.3390/metabo14070356

**Published:** 2024-06-23

**Authors:** Petra Parvanovova, Petra Hnilicova, Martin Kolisek, Zuzana Tatarkova, Erika Halasova, Egon Kurca, Simona Holubcikova, Monika Turcanova Koprusakova, Eva Baranovicova

**Affiliations:** 1 Department of Medical Biochemistry, Jessenius Faculty of Medicine, Comenius University in Bratislava, Mala Hora 4, 036 01 Martin, Slovakia; parvanovova1@uniba.sk (P.P.); zuzana.tatarkova@uniba.sk (Z.T.); sholubcikova@gmail.com (S.H.); 2 Biomedical Centre Martin, Jessenius Faculty of Medicine, Comenius University in Bratislava, Mala Hora 4, 036 01 Martin, Slovakia; petra.hnilicova@uniba.sk (P.H.); martin.kolisek@uniba.sk (M.K.); erika.halasova@uniba.sk (E.H.); 3 Department of Neurology, University Hospital Martin, Jessenius Faculty of Medicine, Comenius University in Bratislava, Kollarova 2, 036 01 Martin, Slovakia; egon.kurca@uniba.sk

**Keywords:** amyotrophic lateral sclerosis, nuclear magnetic resonance, metabolomics, muscle, plasma

## Abstract

Amyotrophic lateral sclerosis (ALS) is a fatal neuromuscular disease type of motor neuron disorder characterized by degeneration of the upper and lower motor neurons resulting in dysfunction of the somatic muscles of the body. The ALS condition is manifested in progressive skeletal muscle atrophy and spasticity. It leads to death, mostly due to respiratory failure. Within the pathophysiology of the disease, muscle energy metabolism seems to be an important part. In our study, we used blood plasma from 25 patients with ALS diagnosed by definitive El Escorial criteria according to ALSFR-R (Revised Amyotrophic Lateral Sclerosis Functional Rating Scale) criteria and 25 age and sex-matched subjects. Aside from standard clinical biochemical parameters, we used the NMR (nuclear magnetic resonance) metabolomics approach to determine relative plasma levels of metabolites. We observed a decrease in total protein level in blood; however, despite accelerated skeletal muscle catabolism characteristic for ALS patients, we did not detect changes in plasma levels of essential amino acids. When focused on alterations in energy metabolism within muscle, compromised creatine uptake was accompanied by decreased plasma creatinine. We did not observe changes in plasma levels of BCAAs (branched chain amino acids; leucine, isoleucine, valine); however, the observed decrease in plasma levels of all three BCKAs (branched chain alpha-keto acids derived from BCAAs) suggests enhanced utilization of BCKAs as energy substrate. Glutamine, found to be increased in blood plasma in ALS patients, besides serving for ammonia detoxification, could also be considered a potential TCA (tricarboxylic acid) cycle contributor in times of decreased pyruvate utilization. When analyzing the data by using a cross-validated Random Forest algorithm, it finished with an AUC of 0.92, oob error of 8%, and an MCC (Matthew’s correlation coefficient) of 0.84 when relative plasma levels of metabolites were used as input variables. Although the discriminatory power of the system used was promising, additional features are needed to create a robust discriminatory model.

## 1. Introduction

Amyotrophic lateral sclerosis (ALS) is a neurodegenerative motor neuron disease leading to death, mostly due to respiratory failure. ALS affects people worldwide, with an incidence of approximately 2/100,000 of the population, more commonly affecting men than women [1,2,3], with onset between 55 and 65 years. Only 5–10% of cases are classified as a familial form with a genetic background. However, the other 90% present a sporadic form. Despite considerable efforts, the causes and pathophysiology of the disease are still poorly understood and complicated by the heterogeneity of the disease. Several cellular processes contributing to ALS manifestation and progress have been identified, such as mitochondrial dysfunction, protein aggregation, free radical formation, excitotoxicity, inflammation, and apoptosis [4,5,6]. Diagnosis of ALS is challenging as there may be a large time window between the first pathological changes and the appearance of symptoms. So far, the diagnostic process is based on history, physical examination, and auxiliary electrodiagnostic methods; however, it is greatly hampered by the absence of unambiguous biochemical or physiological biomarkers that would indicate and expedite the recognition of this disease. ALS is a disease characterized by a gradual onset of symptoms. Initial symptoms of ALS often include difficulties in performing fine motor tasks, leg weakness, and stumbling. With the progress of the disease, motor control of the hands and feet is lost, and speech difficulties may develop, accompanied by non-motor symptoms in about half of ALS patients, more pronounced in patients with bulbar onset [7]. Cognitive decline in ALS is characterized by personality change, irritability, obsessiveness, poor insight, deficits in attention, verbal fluency, and memory, together with behavioral symptoms such as apathy or aggressive behavior in more advanced stages of the disease [8]. Dysfunction of the autonomic nervous system is associated with shorter patient survival: common symptoms include urgency and frequency of urination, gastrointestinal dysfunction (constipation), as well as cardiovascular abnormalities, including reduced heart rate variability [9]. The peripheral sensory impairment worsens concomitantly with motor impairment [10].

There is growing evidence that aberrant energy metabolism could play an essential role in the pathogenesis of ALS [11]. Many studies confirmed mitochondrial dysfunction in ALS patients (reviewed, e.g., [12,13,14]), which seems to be one of the key manifestations of ALS disease. Motor neurons are known to be particularly vulnerable to energetic stress [15], and, in addition, axonal transport is an energy-demanding process that is essential to provide synapses with the necessary proteins and sufficient energy, as well as to mediate signaling back to the cell body and to ensure clearance of detritus [16]. The ALS presence and progression were evident at the metabolic level, where metabolomics changes in the cerebrospinal fluid [17,18] and in the circulation [18,19,20,21,22] were observed. Analysis of the lipidomic profile identified a dysregulated lipid metabolism [23]. The study by Marino et al. [22] reported significant alteration of phosphocholine, lysophosphatidylcholine, and sphingomyelin metabolism, consistent with the exigency of lipid remodeling to repair advanced neuronal degeneration and inflammation in advanced ALS patients.

The endocannabinoid system is involved in multiple aspects of neural function, including motor coordination, learning and memory, emotion, and motivation, and also plays an important role in energy balance. It influences appetite, food consumption, and eating motivation by stimulating exogenic pathways in the hypothalamus. This system may interact with pathophysiological mechanisms at the molecular and cellular level by influencing neuroinflammation, microglial activation, oxidative stress, and excitotoxicity. As ALS is generally characterized by energy imbalance (hypermetabolism, mitochondrial dysfunction, abnormal carbohydrate metabolism, insulin resistance, and dyslipidemia), the agents related to endocannabinoids could be targets for future therapeutic interventions due to their broad effects either in energy metabolism or directly affecting the CNS. The available clinical trials focus more on improving synaptosomes than disease modification; for example, positive effects have improved spasticity, insomnia, and appetite [24].

In the presented work, we used the nuclear magnetic resonance (NMR) method to explore metabolic changes in blood plasma in 25 patients with ALS diagnosed by definitive El Escorial criteria. We employed multivariate PCA and PLS-DA analyses to evaluate differences in metabolome against subjectively healthy controls and compared the results with a cross-validated Random forest discriminatory algorithm to propose and critically discuss the metabolic features that could be considered as metabolic biomarkers of ALS. When considering pathobiology, we focused mainly on alterations in energy metabolism within muscle tissue functioning, such as creatine/creatinine metabolism and branched-chain amino acids transamination with consequent muscle ammonia detoxification. Based on the results, we aimed to describe the biochemical pathways to suggest and communicate the pathophysiology of the ALS disease.

## 2. Materials and Methods

### 2.1. Patient Cohort

Altogether, 25 patients (14 male and 11 female patients), with a median age of 53 ±19 years, were selected for the study. Patients were diagnosed according to the definitive El Escorial criteria. The patients’ ALSFR-S (Revised Amyotrophic Lateral Sclerosis Functional Rating Scale) [25] score was determined at the time of collection; the minimum was 12 points, the maximum was 45 points, and the median of the patient set was 35 points. The diagnosis of the patients included a detailed medical history and physical examination; diseases mimicking ALS were also excluded. Other diseases were excluded based on differential diagnostics (anamnesis, objective neurological examination, complete laboratory parameters, oncomarkers, ancillary investigation methods- electrophysiology, imaging methods- CT- chest, abdomen, pelvis, MRI- brain, spinal canal). All patients definitely met El Escorial criteria and Awaji Shima criteria for ALS disease and progression over time. The blood collections were performed at the neurology clinic of the University Hospital Martin. Patients were informed correctly and signed informed consent before collection. Only patients with compensated chronic diseases were included in the study. Exclusion criteria were age under 18 years and unwillingness or incapability to sign the informed consent. A detailed overview of the patients is given in Table 1.

The study was approved by the Ethical Committee of Jessenius Faculty of Medicine in Martin, Comenius University, Slovakia (Certification code at the US Office for Human Research Protection, US Department of Health and Human Services: IRB00005636 Jessenius Faculty of Medicine, Comenius University in Martin IRB # 1) with identification number: EK43/2021.

Patients had other comorbidities, the most common being arterial hypertension (13 patients), diabetes mellitus (1 patient), myocardial infarction in history (1 patient), ischemic heart disease (1 patient), angina pectoris (1 patient), sinus tachycardia (1 patient), and aortic stenosis (1 patient), stroke (1 patient), multi-infarct encephalopathy (1 patient), transient ischemic attack (1 patient), dementia syndrome (1 patient), fatigue syndrome (1 patient), depressive syndrome (1 patient), anxiety depressive disorder (1 patient), borreliosis (3 patients), breast cancer in remission (2 patients), bronchial asthma (1 patient), bronchitis spastica (1 patient), morbus gravis based (1 patient), hypothyroidism (3 patients), euphunting goiter (1 patient), hypovitaminosis D (1 patient), hypovitaminosis B12 (1 patient), Gilbert’s syndrome (1 patient), hepatic steatosis (2 patients), hepatopathy (2 patients), thrombocytopenia (2 patients), dyslipidaemia (2 patients), hyperlipidaemia (1 patient), hyperlipoproteinaemia (3 patients), anaemia (1 patient), hypercholesterolaemia (1 patient), erythema migrans (1 patient), chronic pancreatitis (1 patient), laryngitis chronica simplex (2 patients). Patients did not have any special diet and did not declare intake of any dietary supplements. The clinical biochemical parameters from patients as well as from the controls are listed in Table 2. The average BMI index was 23.59. Fifteen patients had normal BMI, two were underweight, and eight were slightly overweight. The lowest BMI index was 17.4, and the highest was 29.07, the median was 23.03.

Blood samples from age and gender-matched 25 subjectively healthy volunteers (age difference ± 1 year) from our internal biobank (made from university employees and relatives) having the following features: 24 non-smokers and one person regularly chewing tobacco, not reporting any acute or chronic difficulties/diseases, were used as controls.

### 2.2. Sample Preparation

The peripheral blood was used for analysis. Blood was collected in EDTA-coated tubes, centrifuged at 4 °C, 2000 rpm (380 g-force), for 20 min. Plasma was deproteinized, according to Gowda et al. [26]. The mixture obtained after adding 600 μL of methanol to 300 μL of plasma was shortly vortexed and stored at −20 °C for 20 min. After centrifugation at 14,000 rpm (14,800 g-force), 700 μL of supernatant were dried out. Before measurement, the dried matter was carefully mixed with 100 μL of stock solution and 500 μL of deuterated water. 550 μL of the final mixture was transferred into a 5 mm NMR tube. The stock solution consisted of 100 mM phosphate buffer (pH-meter reading 7.40) and 0.30 mM TSP-d_4_ (3-(trimethylsilyl)-propionic-2,2,3,3-d_4_ acid sodium salt) as a chemical shift reference in deuterated water.

### 2.3. NMR Data Acquisition

NMR data were acquired on 600 MHz NMR spectrometer Avance III from Bruker equipped with TCI (triple resonance) cryoprobe. Initial settings were carried out on an independent sample and adopted for measurements. Samples were stored in Sample Jet at approximately 5 °C before measurement for a maximum of 2 h. We used standard Bruker profiling protocols that we modified as follows: profiling 1D NOESY with presaturation (noesygppr1d): FID size 64 k, dummy scans 4, number of scans 128, spectral width 20.4750 ppm; COSY with presaturation was acquired for randomly chosen ten samples (cosygpprqf): FID size 4 k, dummy scans 8, number of scans 1, spectral width 16.0125 ppm; homonuclearJ-resolved (jresgpprqf): FID size 8 k, dummy scans 16, number of scans 4; profiling CPMG with presaturation (cpmgpr1d, L4 = 126, d20 = 3 ms): FID size 64 k, dummy scans 4, number of scans 128, spectral width 20.0156 ppm. All experiments were conducted with a relaxation delay of 4 s; all data were once zero-filled. An exponential noise filter was used to introduce 0.3 Hz line broadening before the Fourier transform. Samples were measured at 310 K and randomly ordered for acquisition.

### 2.4. Data Analysis

A chemical shift of 0.000 ppm was assigned to the TSP-d_4_ signal. All spectra were binned in bins of the size of 0.001 ppm, starting from 0.500 ppm to 9.500 ppm. No normalization method was applied to NMR data, as we took the same amount of blood plasma from all samples for the analysis. Spectra were solved using an internal metabolite database, an online human metabolome database (www.hmdb.ca, accessed 1–2 March 2024) [27], chenomx software (NMR suite 9.0) free trial version, and literature [26]. For all compounds, the multiplicity of peaks was confirmed in J-resolved spectra, and homonuclearcross-peaks were confirmed in COSY spectra. After identifying the metabolites, we chose the spectra subregions with only a single metabolite assigned. In 0.001 ppm binned spectra, we summed integrals of selected signals. These values were used as relative concentrations of metabolites in blood plasma. Metabolites that did not have appropriate signals for the evaluations, or their peak assignment was not unambiguous, were excluded from further processing.

The null hypothesis of equality of population medians between groups was tested by the non-parametric Mann-Whitney U test (GraphPad). Principal component analysis (PCA), sparse partial least squares discriminant analysis (sPLS-DA), and the receiver operating characteristic curves (ROC) derived from the cross-validated random forest (RF) algorithm were performed in Matlab (2018b) and online tool Metaboanalyst 6.0 [28].

Note: In this work, we use common labeling BCAAs for branched-chain amino acids: leucine, isoleucine, and valine, and BCKAs for their 2-oxo derivates, branched-chain keto acids: ketoleucine (2-oxoisocaproate), ketosioleucine (3-methyl-2-oxopentanonate) and ketovaline (2-oxoisovalerate), as well as mentioned trivial names of BCKAs.

## 3. Results

In this work, following metabolites were considered: lactate, BCAAs (leucine, isoleucine, valine), BCKAs (ketoacids from leucine, isoleucine, valine), alanine, glucose, pyruvate, succinate, citrate, phenylalanine, tryptophan, tyrosine, creatine, creatinine, glutamine, lysine, histidine, proline 3-hydroxybutyrate and lipoprotein fraction consisting up to 40% from triacylglycerols [29]. Lipoprotein fraction, not meeting the criteria of metabolite, was excluded from multivariate analyses since it also includes the contributions from very low-density lipoprotein (VLDL), low-density lipoprotein (LDL), and high-density lipoprotein (HDL) [29].

**Figure 1:**
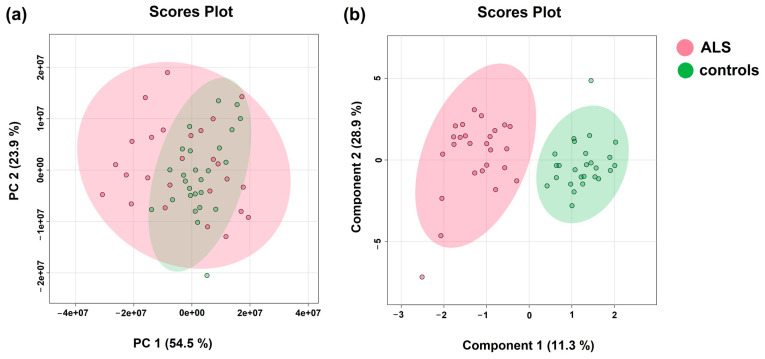
(**a**) PCA analysis(Principal component analysis); (**b**) sPLSDA analysis (Sparse Partial Least Squares Discriminant Analysis) of patient groups (ALS) and controls; relative levels of blood plasma metabolites determined by NMR were used as input variables.

Multivariate PCA analysis serves to visualize multidimensional metabolic data in 2D space using a smaller set of variables known as principal components that are linear combinations of the original variables. The two major principal components (PC1 and PC2) are the two directions in space along which the data sets have the highest or most variance. This method often serves as the first step when accessing metabolic data. Based on the data gained in this study, the metabolic profiles of ALS patients in blood plasma were clustered within a larger confidence interval (95%) than those from controls, suggesting greater data variability among samples (Figure 1 left, PCA). (The inclusion of parameters from clinical biochemistry to metabolite levels did not improve the PCA performance. When employing the multivariate sPLS-DA procedure, which also includes a discriminatory algorithm, the clusters formed from ALS patients’ data and those from controls separated, not showing overlap within a 95% confidence interval (Figure 1). These visualizations suggest that the discrimination between ALS and controls, based on levels of blood plasma metabolites, could be attainable. The most important features resulting from sPLSDA, which were responsible for discrimination, were creatinine, creatine, glutamine, and BCKAs. However, PLS-DA-based algorithms are known to severely overfit data [30], leading to overoptimistic results. To detect potential overfitting of the current sPLS-DA model, we added the 10-fold cross-validation, which should avoid generalizing the pattern from unsubsampled data. The performance of the 10-fold CV sPLS-DA model was expressed by the error rate in the range of 7.7–15.4%.

To obtain a more realistic estimation of discriminatory performance, we decided on the Monte-Carlo cross-validated RF classification [31] to get a realistic estimate of the discriminative performance. The area under the curve (AUC) derived from the receiver operating characteristic curve (ROC), OOB (out of bag) error, and Matthews correlation coefficient (MCC) [32] were used as parameters for quantitative accessing the discriminatory power of the system.

**Figure 2:**
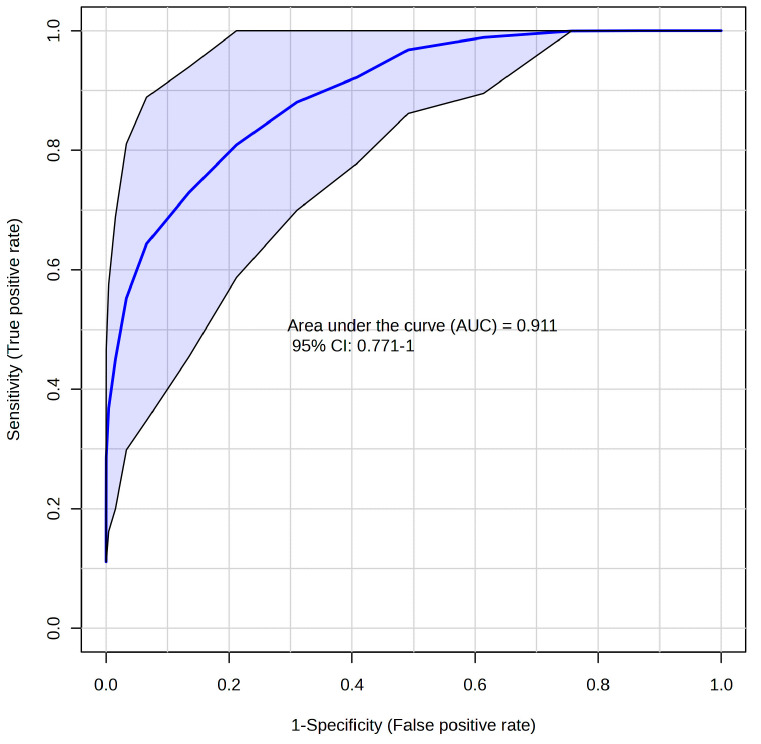
ROC curve based on Random Forest algorithm for discrimination of ALS—controls; relative levels of blood plasma metabolites determined by NMR were used as input variables.

When discriminating patients against controls via cross-validated Random Forest, we obtained a promising result when the algorithm finished with an OOB error of 0.11, AUC = 0.911 (Figure 2). However, MCC, which is considered a more reliable criterion than AUC and OOB error, suggests a weaker discrimination power with an obtained value of 0.84. The most important features responsible for discrimination against controls were marked creatinine, creatine, and BCKAs. These variables were evaluated individually to test their discrimination power as single predictors. The results are listed in Table 3 in detail.

**Figure 3:**
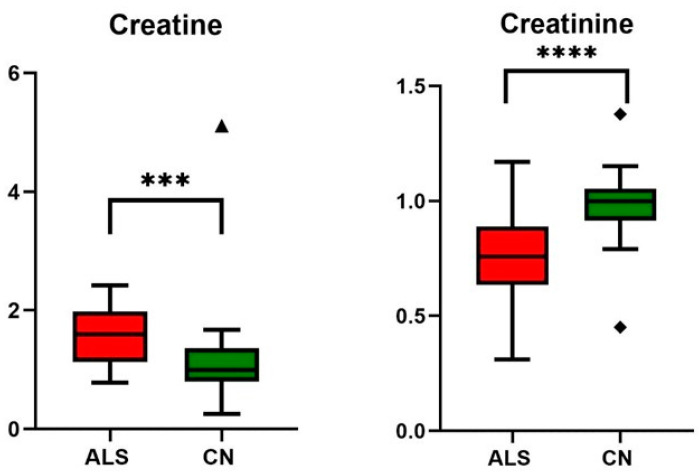
Relative changes in blood plasma creatine and creatinine in ALS patients vs. controls (CN); data were normalized to the median of CN set to 1; *** *p*-value < 0.0005 and **** *p*-value < 0.0001, symbols (▲ and ◆) are used to show the outliers.

**Figure 4:**
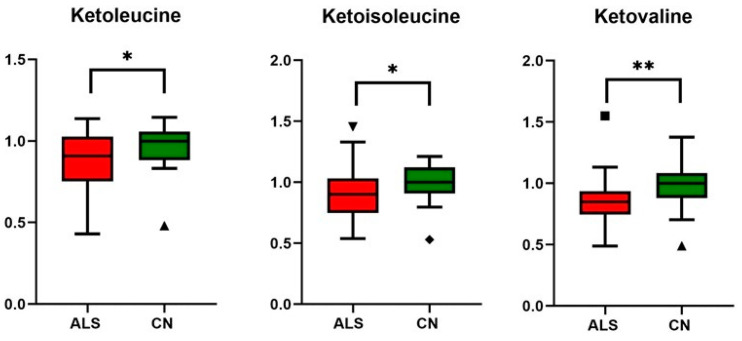
Relative changes in blood plasma BCKAs (branched chain keto acids) in ALS patients vs. controls (CN); data were normalized to the median of CN set to 1; * *p*-value < 0.05 and ** *p*-value < 0.005, symbols (▲ and ◆) are used to show the outliers.

**Figure 5:**
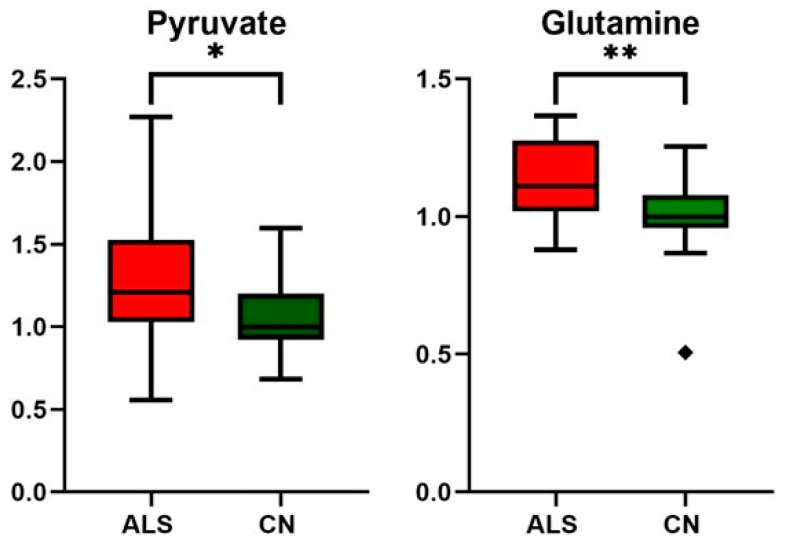
Relative changes in blood plasma pyruvate and glutamine in ALS patients vs. controls (CN); data were normalized to the median of CN set to 1; * *p*-value < 0.05 and ** *p*-value < 0.005, symbol ◆ is used to show the outlier.

In the next part, we focused on gaining an improved biological understanding of ALS disease by exploring the metabolite profiles. From the data obtained, significant differences were found in metabolites included in energy metabolism: pyruvate, creatine, creatinine, BCKAs, and glutamine when considering the standardly used threshold to claim significance—α level of 0.05 (Table 4, Figure 3, Figure 4 and Figure 5). It should be noted that when using small sample sets (*n* = 25 for each group), the statistical power of the test is low, with a high probability of committing Type II error, which is a false negative observation that some features (metabolite levels) would not be recognized as different, although they may be in real.

## 4. Discussion

ALS is an adult-onset motor neuron disorder characterized by progressive motor symptoms, such as muscle weakness, muscle atrophy, and spasticity. Muscle malfunctioning is also evident from elevated creatine kinase in blood over the reference range (Table 2), an observation frequently detected in ALS patients indicating muscle cell breakdown and damage [33]. For proper muscle function, one of the most critical energy-gaining processes is the creatine intake from circulation, conversion to phosphocreatine, and energy release with creatinine as a waste product excluded from the blood by the renal way. A significant increase in blood creatine levels accompanied by a decrease in circulating creatinine level was already observed in ALS patients with advanced disease previously [18,22,34,35]. Our observations were very similar for both creatine and creatinine as determined by the NMR method (Table 4, Figure 3), as well as for creatinine through clinical biochemistry (Table 2). It seems that in times of progressive muscle denervation with muscle wasting, the ability of muscle tissue to take up creatine from circulation is limited, and creatine may accumulate in the blood. As a result, creatinine production is restricted, showing lowered blood levels. Some studies were conducted on ALS patients supplemented by creatine monohydrate to improve muscle activity and prolong the life of patients. Although creatine monohydrate was well tolerated, it did not have an obvious benefit on the multiple markers of disease progression [36,37,38]. Based on this knowledge and the results from recent metabolic studies, increasing already high creatine levels in circulation does not appear to be relevant support since improper muscle functioning in ALS patients is likely linked with the insufficiency of muscles to utilize creatine for energy production.

When evaluating blood plasma amino acids levels, we did not detect any tendency for shortage or excess of BCAA or other proteogenic amino acids phenylalanine, tyrosine, tryptophane, histidine, and proline, in ALS patients, which is a sign of their balanced pool in circulation. ALS patients generally show accelerated skeletal muscle catabolism [39]. ALS subjects in this study showed a decreased amount of total protein (Table 2) in blood plasma compared with controls, with the median at the lower limit of the reference range. Protein breakdown could lead to the accumulation of amino acids unless they would be utilized otherwise, most likely as an energy source [40]. In fact, we did not observe any tendency of blood urea levels to increase, one of the end products of protein and amino acids catabolism, in ALS patients against controls (Table 2). Based on this, we could conclude that the rate of proteolysis seems to be of that value on which the body manages to balance the released amount of amino acids over the time period without accumulation of intermediates or waste products catchable by routine clinical biochemistry.

**Figure 6:**
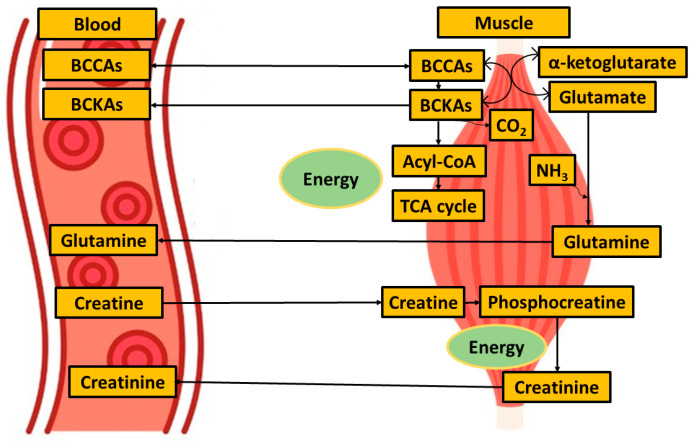
Simplified metabolites exchange between blood and muscle when focused on muscle energy metabolism.

Although BCAAs plasma levels were unaltered, we observed decreased plasma levels of all three BCKAs (ketoleucine, ketoisoleucine, and ketovaline, Table 4, Figure 4). When compared with other studies, these alpha-keto acids plasma levels have not yet been evaluated in ALS conditions, as they are often not covered by untargeted metabolic approaches. BCKAs are produced by reversible deamination of BCAAs. Simplified, BCAAs are transaminated by BCAT (BCAA transaminase) to generate glutamate, which, after deamination to α-ketoglutarate, anaplerotically enters the TCA cycle and produces ammonia [41]. BCKAs, if not released from muscle into the bloodstream, are irreversibly decarboxylated to yield respective CoA compounds, which, after particular metabolic pathways, may be used as substrates in the TCA cycle. In our study, besides a decrease in BCKAs, we observed significantly increased levels of circulating glutamine, consistent with the accelerated utilization of amino acids as an energy substrate with subsequent muscle ammonia detoxification through glutamine (schematic Figure 6). Glutamine is, under normal conditions, maintained within skeletal muscle in high concentration, produced from ammonia and glutamate, a reaction catalyzed by glutamine synthetase, which was found to be over-expressed in ALS patients in serum as well as in cerebrospinal fluid [42]. The data also provide evidence about downregulated BCAT expression in cellular models of sarcopenia [43]. However, ALS mimics conditions or experimental models, and data that could enrich the discussion are not known. The increase in BCKAD (branched-chain α-ketoacid dehydrogenase enzyme complex, catalyzing the irreversible oxidative decarboxylation of BCKA needed to use BCKAs as energy substrate) activity localized in muscles was demonstrated previously in protein catabolic states [44], which support the thesis of accelerated amino acids utilization as energy substrates.

Even though BCAAs are acknowledged as essential for good muscle health, supporting treatment of ALS by BCAAs supplementation failed in the study in 1993 [45] as well as later in the study by Tandan [46] and Testa [47]. A few decades later, the excessive consumption of BCAAs was even considered a potential contributor to the development of neurodegeneration disorders, including ALS [48], which was supported by experimental studies on mice [49]. So far, no study has been completed on the supplementation with BCKAs, which are, based on our results, decreased in ALS patients in circulation and potentially could support the organism in energy-gaining metabolism. Based on the data obtained, BCKAs could be an essential contributor to muscle energy metabolism when the energy-gaining process from the creatine pathway is suppressed in ALS patients. However, whether the muscles or other tissues would be able to metabolize more BCKAs, if provided, is not clear. Further, caution is necessary, while the alpha-keto analogs of BCAAs can also cross the blood-brain barrier [50], and the complementation may disrupt competition among circulating metabolites crossing BBB.

Although the pathogenesis of ALS remains unclear, increasing evidence suggests that a key contributing factor is mitochondrial dysfunction [13]. Based on results from other studies, as well as what we observed, glucose metabolism seems not to be affected in ALS patients, as both blood glucose and lactate levels did not show alterations (Table 4). As shown in the study by Hui et al. [51], the contribution of glucose to tissue TCA metabolism is primarily indirect (via circulating lactate) in all tissues except the brain. In other words, tissue uses glucose to produce lactate, which is released into the bloodstream, re-uptaken by other tissue, converted to pyruvate, and used as a TCA substrate. Pyruvate, to be used as an energy substrate, should first cross the mitochondrial barrier, and its conversion to acetyl-Co-A is set up within the mitochondria. In ALS individuals, we detected increased blood pyruvate levels (Table 4, Figure 6), and the data suggest that either the pyruvate entry into or the utilization of pyruvate within mitochondria is, in relation to current knowledge, most likely affected.

Interestingly, the blood plasma levels of TCA intermediates citrate and succinate did not show any changes in ALS patients against controls (Table 4). This suggests that the TCA cycle is ongoing, and in the conditions of limited pyruvate utilization, TCA is maintained by other sources. One possibility of preserving the TCA cycle is the enhanced utilization of lipids, which would be manifested in the increased amount of 3-hydroxybutyrate, a ketone body representative, which was not observed in this study in ALS patients. Therefore, as a potential precursor for anaplerotic reactions, we could consider glutamine, which is present in sufficient amounts in circulation.

### Multivariate Analyzes

Current metabolic studies are aimed at two general questions: (i) to gain improved biological understanding through the analysis of metabolite profiles accompanied by *p*-value from hypothesis testing, and (ii) biomarker analysis, requiring different analysis, evaluation, and validation, such as supervised machine learning algorithms, or multivariate regression models which should be used to build the predictive models needed for biomarker analysis. Biomarkers are designed to discriminate with an optimal sensitivity/specificity without regard to biological cause or biological interpretation. Significant differences in the metabolite levels between groups do not guarantee that the compounds will be good classifiers, and otherwise, metabolites that are not significant in isolation can, when combined into a multivariate model, produce reproducible discrimination [52].

The potential of metabolites to serve as biomarkers for ALS was described in previous studies. Chang et al. obtained an AUC of 0.945 after the SVM algorithm with an accuracy of 0.88 after cross-validation when using plasma levels of phosphatidylcholines, creatinine, and methionine [53]. Using logistic regression, Jie et al. [54] obtained AUC values over 0.96 for metabolites determined by GC/MS metabolomics assay. In this study, we investigated the discriminatory power of the system ALS patients vs. healthy controls when relative levels of blood metabolites as determined by NMR were used as input variables. Firstly, we employed sPLS-DA, which resulted in well-separated clusters from ALS patients and controls. For better performance estimation, we included 10-fold cross-validation into the algorithm, which finished with an error of 7.7–15.4% (random sub-sampling in repeated runs, especially in small data sets, can cause slight variation). This result highlights the need for validation when discriminating the classes, as without this, the algorithm is fitted only to the data given and may not work successfully on the new data. The metabolic features determined as most important for sPLSDA discrimination were creatinine, creatine, glutamine, and BCKAs.

In the next step, we used a cross-validated Random Forest discriminatory algorithm, which is not known to overfit the data and gives more reliable results. The RF run finished with an AUC of around 0.70 for individual BCKAs; slightly better was the performance for creatine or creatinine, with an AUC over 0.82 (Table 3). The best performance was achieved using all these features with 3-hydroxybutyrate, glutamine, and pyruvate, namely AUC over 0.911 with 8% OOB error and MCC of 0.84 (Table 3). As Lopez et al. [55] described, we cannot define the metabolites as biomarkers only based on an ROC without clinical validation. To create the ROC curve, we used the RF algorithm, which picks up two-thirds of the data for training, the rest for testing for regression, and almost 70% of the data for training and the rest for testing during classification in order to overcome the training and testing on the same data. Although this approach does not substitute clinical validation, it may lead to encouraging results in exploratory studies. The discriminatory power of the system used is promising but not ideal, and some additional features are needed to create a robust discriminatory model.

Notes:

(i) Our study had some limitations. Due to the rarity of ALS, we used a small patient cohort. The diagnosis of this disease is challenging, with an average diagnostic delay of one year. The patients included in this study have full-blown disease, and the rate of progression and length of survival with this disease is mainly individual. We also cannot determine the impact of medication on metabolic changes.
(ii) NMR metabolomics covers the relatively small number of endogenous molecules related to the number of genes, RNA species, or proteins. It is obvious that in the identification of potential low molecular biomarkers, some overlap between different pathologies can occur. Therefore, and also based on the biology described above, we cannot exclude that the molecules found in this work as potential biomarkers in combination overlap with other disorders, including muscle atrophy and spasticity.
(iii) The injury of one organ can lead to compensatory effects or secondary injury. Although the observed metabolomic changes in plasma could be ascribed to the muscle energy metabolism, the systematic metabolic finding is always the result of the complex mutual biochemical pathways in the comprehensive inter-organ metabolic exchange and communication.

## 5. Conclusions

ALS patients included in this study showed increased levels of creatine and decreased levels of creatinine in circulation, which is in line with the condition of limited utilization of creatine for energy production by muscles. This finding could explain the failure of many studies to improve the energy muscle metabolism by creatine monohydrate supplementation. The accelerated proteolysis in ALS patients seems to be of that value on which the body manages to balance the released amount of amino acids over the time period without accumulation of amino acids or waste products catchable by routine clinical biochemistry or by NMR metabolomic approach. The fact that ALS patients do not suffer from amino acid deficiency makes further supplementation by amino acids irrelevant. Based on the data obtained, BCKAs could be an essential contributor to muscle energy metabolism when the energy-gaining process from the creatine pathway is suppressed. However, the ability of muscles or other tissues to metabolize more BCKAs, if provided in a condition of ALS or mimicking ALS, has not been tested so far.

The discrimination of binary system ALS patients vs. healthy controls by cross-validated Random Forest was promising when the algorithm was fed relative levels of plasma metabolites as determined by NMR. The most important metabolites were determined: creatine, creatinine, BCKAs, glutamine, and pyruvate. However, the obtained values, AUC over 0.91 with 8% OOB error and MCC of 0.84 suggest the need for additional features to build a more robust model. NonethelessBesides, we cannot exclude that the molecules found in this work as potential biomarkers in combination cannot cover other disorders, mainly related to muscle atrophy, loss, and spasticity.

## Figures and Tables

**Table 1:** Characteristics of patient cohort: diagnostic delay—time from the appearance of the first symptoms to a definitive diagnosis; disease duration—time from the first symptoms of the disease to the measurement of mitochondrial respiration; ΔFS ratio (rate of disease progression); calculated as the rate of disease progression ΔFS = (ALSFR-S total—ALSFR-S actual)/the duration of the disease from the onset of symptoms. Medications: Milgamma N- benfotiamine (40 mg), pyridoxinium chloride (90 mg), and cyanocobalamin (0.25 g).

	Gender	Age	Diagnostic Delay (Months)	Duration (Month)	ALSFRS Score	ΔFS	Medication	Symptoms	Onset of Symptoms
1	male	42	7	7	42	0.85	Riluzole Coenzyme Q10 Milgamma N	Left lower limb weakness, stiffness, motor problem, spastic-paretic gait	Spinal
2	female	50	13	13	43	0.38	Riluzole Coenzyme Q10	Difficulty speaking, dry mouth in the evening and at night, swollen tongue, dysarthric, nasolabial speech	Bulbar
3	male	43	38	38	38	0.26	No medication	Difficulty speaking, dry mouth in the evening and at night, swollen tongue, dysarthric, nasolabial speech	Spinal
4	female	63	12	12	33	1.25	No medication	Gradually progressive weakness of the limbs, predominantly on the left, clumsiness, problems with walking	Spinal
5	female	68	23	23	35	0.56	Milgamma N	Mixed quadriparesis	Spinal
6	male	81	9	9	33	1.6	No medication	Weakness of the right lower limb, gradually also of the left lower limb, generalized fasciculations on the trunk limbs, dysphonia	Spinal
7	female	74	7	7	35	1.85	No medication	Impaired speech, difficulty swallowing, problems with fine motor skills	Bulbar
8	male	60	16	16	41	0.43	No medication	Weakness in the proximal muscles of the left upper limb	Spinal
9	male	54	36	36	37	0.30	No medication	Speech impairment, weakness, and wasting of upper limb muscles are more pronounced on the left	Spinal
10	male	67	20	20	28	1	No medication	Weakness of the left upper and lower limbs; walking with support	Spinal
11	female	54	15	16	22	1.62	Riluzole	Progressive weakness of all limbs, more left side	Spinal
12	male	68	15	15	38	0.6	No medication	Muscle twitching in the upper and lower limbs, progressive weakness of the upper limbs	Spinal
13	female	72	12	12	31	1.41	No medication	Weakness of the arm muscles with predominance on the right, weakness of the legs—only a short walk	Spinal
14	male	62	17	17	38	0.58	No medication	Mixed quadriparesis of moderate severity with marked muscle atrophy accentuated distally in the upper limbs, generalized fasciculations	Spinal
15	female	69	4	4	45	0.75	No medication	Progressive deterioration of articulation, progressive swallowing difficulties and weight loss, mixed paresis of the upper and lower limbs	Bulbar
16	male	71	12	12	35	1.08	No medication	Weakness of the upper limbs, especially in the shoulder muscles, is gradually associated with impaired swallowing and articulation	Spinal
17	female	46	8	8	39	1.12	Milgamma N	Weakness and stiffness of the lower limbs	Spinal
18	female	50	9	26	12	1.0	Riluzole	Immobile without the possibility of verbal communication	Spinal
19	Male	78	12	12	39	0.75	No medication	Weakness of the lower limbs, progressive weakness of the upper limbs, muscle wasting	Spinal
20	Male	60	10	18	30	1.0	Riluzole Coenzyme Q10	Upper limb weakness, significant lower limb weakness, wheelchair mobility	Spinal
21	female	64	13	13	37	0.84	Riluzole Coenzyme Q10 Milgama N	Monoparesis of the right upper limb	Spinal
22	female	75	11	11	37	1.0	No medication	Problem with speech, articulation, difficulty swallowing	Bulbar
23	male	59	12	16	29	1.58	Riluzole	Weakness of the right upper limb, gradually also weakness of the right lower limb, breathing problems	Spinal
24	male	68	11	11	35	1.18	No medication	Speech problems, difficulty swallowing, fine motor problems	Bulbar
25	male	57	12	15	30	1.5	Riluzole Coenzyme Q10	Upper limb weakness, lower limb weakness, difficulty breathing	Spinal

**Table 2:** Standard biochemical and hematological parameters of patients and controls at the sampling times with statistical comparison; #—values deviating from the normal range; IQR—the interquartile range, significant differences in concentrations are shown in bold.

		ALS Patients				Control Subjects, N = 25		*p*-Value
Biochemical Parameters with the Reference Range	N Total/N Missing	Median	IQR	Minimum	Maximum	Median	IQR	
Na mmol/L (136–146)	25/1	140.8	2.7	130	143	138	2	**0.0062**
Cl mmol/L (101–109)	25/2	105	4	90	108	103	2	**0.0057**
K mmol/L (3.5–5.1)	25/2	4.2	0.53	3.4	4.8	4.1	0.3	0.6399
CK (male ≤ 2.85 µkat/L female ≤ 2.42 µkat/L)	25/8	3.37 #	7.985	0.98	19.04			
GMT (male: <0.92 µkat/L female: <0.63 µkat/L)	25/4	0.43	0.725	0.15	4.63	0.29	0.22	0.1139
Albumin g/L (35–52)	25/4	37.7	3.4	20.56	42.3			
Total protein g/L (66–83)	25/5	65.65	1.35	57.7	71.1	71.9	4.5	**<0.0001**
Total bilirubin umol/(5–21)	25/2	12.4	4.72	6.9	36.3	11.2	7.5	0.3644
AST ukat/L (0.1–0.85)	25/4	0.5	0.275	0.22	1.12	0.37	0.06	**0.0021**
ALT ukat/L (0.1–0.85)	25/1	0.53	0.4275	0.17	1.9	0.3	0.18	**0.0014**
ALP ukat/L (0.5–2.15)	25/8	0.96	0.295	0.71	2.12			
Creatinine umol/L (59–104)	25/1	60.63	27.75	6.8	77	74	15	**<0.0001**
Urea mmol/L (2.8–7.2)	25/2	4.8	2.5	1.6	8.2	4.7	1.3	0.8179
Glucose mmol/L (4.1–5.9)	25/1	5.05	0.95	4.3	12.4	5.1	0.4	0.8000
CRP mg/L (0–5)	25/7	1.8	6	0.3	68.4	1.6	2.6	0.4239
WBC (4–10 × 10^9^)	25/1	6	2.325	3.4	10.3	5.18	2.41	0.3478
RCB (4.1–6 × 10^12^)	25/1	4.625	0.558	3.18	5.74	4.75	0.59	0.1613
Hemoglobin g/L (140–179)	25/1	137 #	18.5	95	171	144	16	0.0707
HCT (0.4–0.5)	25/1	0.41	0.0475	0.29	0.51	40.4	5.3	**<0.0001**
MCV (82–89 fL)	25/1	90.7 #	4.17	82.1	96.8	86.5	4.7	**0.0007**
MCH (28–34 pg)	25/1	30.55	2	27.4	32.5	30.5	1.5	0.8003
MCHC (320–360 g/L)	25/1	335	12.3	33	355	352	7	**<0.0001**
RDW (10–15.2%)	25/1	13.65	1.28	12.3	15.7	11.9	4.2	**<0.0001**
PLT (150–400 × 10^9^)	25/1	191	75.7	115	352	251	66	**0.0048**
MPV (7.8–12.8 fL)	25/1	9.05	1.325	6.8	10.9	8.9	1.4	0.9723
PDW (9–17 fL)	25/1	16.85	1.05	15.3	18.2	16.4	0.6	0.1149
Fibrinogen (1.8–3.5 g/L)	25/4	3.35	1.325	2.58	5.63			
PT (75–120%)	25/5	92	8.75	67	108			
INR (0.8–1.2)	25/4	1.05	0.09	0.95	1.32			
APTT (23–35 s)	25/5	30	2.77	22.7	31.7			
APTTr (0.84–1.2)	25/5	1.03	0.09	0.78	1.09			
TT (<21 s)	25/5	14.5	1.5	11.5	17.1			
Neutrofiles (1.4–6.5 × 10^9^/L)	25/8	3.75	2.485	1.46	6.9	2.79	1.11	**0.0427**
Lymphocytes (1.2–3.4 × 10^9^/L)	25/8	1.8	0.51	1.3	3.54	1.85	0.75	0.6254
Eosinofiles (0.05–0.25 × 10^9^/L)	25/8	0.11	0.115	0.06	0.175	0.13	0.13	**0.0360**
Monocites(0.3–0.5 × 10^9^/L)	25/8	0.5	0.335	0.33	0.98	0.36	0.15	**0.0009**
Basofiles (0.0–0.1 × 10^9^/L)	25/8	0.03	0.02	0	0.04	0.02	0.01	0.2320
Neutrofiles % (60–70)	25/8	60.1	13.8	43.4	72.6	55.60	12	0.1173
Lymphocytes % (20–30)	25/8	29.1	11.8	18	44.1	33.2	12.8	**0.0469**
Eosinofiles % (0–5)	25/8	1.7	1.6	0.5	3.3	2.8	1.8	**0.0007**
Monocites % (0–1)	25/8	7.9 #	3.5	5.9	12.8	6.6	2.9	**0.0057**
Basofiles % (0–1)	25/8	0.4	0.4	0.2	0.9	0.4	0.2	0.5284

**Table 3:** Results from the cross-validated Random Forest discrimination for system ALS patients—controls; relative levels of blood plasma metabolites were used as input variables; AUC—area under the curve—related to ROC; OOB—out of bag error; MCC—Mathews correlation coefficient; and BCKAs—branched-chain keto acids.

Variable Used for Discriminatory Performance	AUC	OOB	MCC
Creatinine	0.85	0.12	0.76
Creatine	0.81	0.12	0.76
Glutamine	0.75	0.18	0.64
Ketoleucine	0.72	0.18	0.64
Ketoisoleucine	0.67	0.20	0.60
Ketovaline	0.72	0.18	0.64
Creatinine, Creatine	0.86	0.12	0.76
Creatinine, Creatine, Glutamine, BCKAs, 3-hydroxybutyrate, Pyruvate	0.92	0.08	0.84

**Table 4:** Relative levels of blood plasma metabolites in ALS patients; data were normalized to the median of controls set to 1; *p*-values wereas derived from the Mann–Whitney U test; and change was calculated from medians.

Metabolite	*p*-Value	The Relative Level of a Metabolite in ALS against Control	Change
Lactate	0.2237	1.394	0.394
Alanine	0.8626	0.9997	−0.0003
Valine	>0.9999	0.9983	−0.0017
Glucose	0.1698	1.022	0.022
Leucine	0.4526	0.9260	−0.074
Isoleucine	0.7292	0.9125	−0.0875
Pyruvate	**0.020**	1.208	0.208
Citrate	0.441	1.015	0.015
Phenylalanine	0.6581	0.9939	−0.0061
Tyrosine	0.8026	1.016	0.016
Glutamine	**0.0019**	1.111	0.111
Lysine	0.335	1.019	0.019
Lipoproteins	0.1035	0.9035	−0.0965
3-OH-butyrate	0.6721	1.052	0.052
Ketoleucine	**0.017**	0.9079	−0.0925
Ketoisoleucine	**0.0353**	0.9018	−0.0982
Ketovaline	**0.008**	0.8479	−0.1521
Creatine	**0.0002**	1.599	0.599
Creatinine	**<0.0001**	0.7575	−0.2425
Proline	0.1522	1.028	0.028
Histidine	0.4185	0.9252	−0.0748
Succinate	0.1888	0.9024	−0.0976
Tryptophan	0.0994	0.8901	−0.1099
Glycine	0.2389	1.213	0.213

Significant differences in concentrations are shown in bold.

## Data Availability

Raw NMR spectra, as well as the data evaluated, are available upon request from Eva Baranovičová.

## References

[1] Longinetti E., Fang F. (2019). Epidemiology of Amyotrophic Lateral Sclerosis: An Update of Recent Literature. Curr. Opin. Neurol..

[2] McCombe P.A., Henderson R.D. (2010). Effects of Gender in Amyotrophic Lateral Sclerosis. Gend. Med..

[3] Manjaly Z.R., Scott K.M., Abhinav K., Wijesekera L., Ganesalingam J., Goldstein L.H., Janssen A., Dougherty A., Willey E., Stanton B.R. (2010). The Sex Ratio in Amyotrophic Lateral Sclerosis: A Population Based Study. Amyotroph. Lateral Scler..

[4] Salucci S., Bartoletti Stella A., Battistelli M., Burattini S., Bavelloni A., Cocco L.I., Gobbi P., Faenza I. (2021). How Inflammation Pathways Contribute to Cell Death in Neuro-Muscular Disorders. Biomolecules.

[5] Michalska P., León R. (2020). When It Comes to an End: Oxidative Stress Crosstalk with Protein Aggregation and Neuroinflammation Induce Neurodegeneration. Antioxidants.

[6] Gordon P.H. (2011). Amyotrophic Lateral Sclerosis: Pathophysiology, Diagnosis and Management. CNS Drugs.

[7] Chiò A., Moglia C., Canosa A., Manera U., Vasta R., Brunetti M., Barberis M., Corrado L., D’Alfonso S., Bersano E. (2019). Cognitive Impairment across ALS Clinical Stages in a Population-Based Cohort. Neurology.

[8] Crockford C., Newton J., Lonergan K., Chiwera T., Booth T., Chandran S., Colville S., Heverin M., Mays I., Pal S. (2018). ALS-Specific Cognitive and Behavior Changes Associated with Advancing Disease Stage in ALS. Neurology.

[9] Dubbioso R., Provitera V., Pacella D., Santoro L., Manganelli F., Nolano M. (2023). Autonomic Dysfunction Is Associated with Disease Progression and Survival in Amyotrophic Lateral Sclerosis: A Prospective Longitudinal Cohort Study. J. Neurol..

[10] Nolano M., Provitera V., Caporaso G., Fasolino I., Borreca I., Stancanelli A., Iuzzolino V.V., Senerchia G., Vitale F., Tozza S. (2024). Skin Innervation across Amyotrophic Lateral Sclerosis Clinical Stages: New Prognostic Biomarkers. Brain.

[11] Vandoorne T., De Bock K., Van Den Bosch L. (2018). Energy Metabolism in ALS: An Underappreciated Opportunity?. Acta Neuropathol..

[12] Stanga S., Caretto A., Boido M., Vercelli A. (2020). Mitochondrial Dysfunctions: A Red Thread across Neurodegenerative Diseases. Int. J. Mol. Sci..

[13] Zhao J., Wang X., Huo Z., Chen Y., Liu J., Zhao Z., Meng F., Su Q., Bao W., Zhang L. (2022). The Impact of Mitochondrial Dysfunction in Amyotrophic Lateral Sclerosis. Cells.

[14] Genin E.C., Abou-Ali M., Paquis-Flucklinger V. (2023). Mitochondria, a Key Target in Amyotrophic Lateral Sclerosis Pathogenesis. Genes.

[15] Harris J.J., Jolivet R., Attwell D. (2012). Synaptic Energy Use and Supply. Neuron.

[16] Sheng Z.-H. (2017). The Interplay of Axonal Energy Homeostasis and Mitochondrial Trafficking and Anchoring. Trends Cell Biol..

[17] Gray E., Larkin J.R., Claridge T.D.W., Talbot K., Sibson N.R., Turner M.R. (2015). The Longitudinal Cerebrospinal Fluid Metabolomic Profile of Amyotrophic Lateral Sclerosis. Amyotroph. Lateral Scler. Front. Degener..

[18] Wu J., Wuolikainen A., Trupp M., Jonsson P., Marklund S.L., Andersen P.M., Forsgren L., Öhman A. (2016). NMR Analysis of the CSF and Plasma Metabolome of Rigorously Matched Amyotrophic Lateral Sclerosis, Parkinson’s Disease and Control Subjects. Metabolomics.

[19] Germeys C., Vandoorne T., Bercier V., Van Den Bosch L. (2019). Existing and Emerging Metabolomic Tools for ALS Research. Genes.

[20] Kumar A., Bala L., Kalita J., Misra U.K., Singh R.L., Khetrapal C.L., Babu G.N. (2010). Metabolomic Analysis of Serum by (1) H NMR Spectroscopy in Amyotrophic Lateral Sclerosis. Clin. Chim. Acta.

[21] Goutman S.A., Boss J., Guo K., Alakwaa F.M., Patterson A., Kim S., Savelieff M.G., Hur J., Feldman E.L. (2020). Untargeted Metabolomics Yields Insight into ALS Disease Mechanisms. J. Neurol. Neurosurg. Psychiatry.

[22] Marino C., Grimaldi M., Sommella E.M., Ciaglia T., Santoro A., Buonocore M., Salviati E., Trojsi F., Polverino A., Sorrentino P. (2022). The Metabolomic Profile in Amyotrophic Lateral Sclerosis Changes According to the Progression of the Disease: An Exploratory Study. Metabolites.

[23] Goutman S.A., Guo K., Savelieff M.G., Patterson A., Sakowski S.A., Habra H., Karnovsky A., Hur J., Feldman E.L. (2022). Metabolomics Identifies Shared Lipid Pathways in Independent Amyotrophic Lateral Sclerosis Cohorts. Brain.

[24] Urbi B., Broadley S., Bedlack R., Russo E., Sabet A. (2019). Study Protocol for a Randomised, Double-Blind, Placebo-Controlled Study Evaluating the Efficacy of Cannabis-Based Medicine Extract in Slowing the Disease pRogression of Amyotrophic Lateral Sclerosis or Motor Neurone Disease: The EMERALD Trial. BMJ Open.

[25] Cedarbaum J.M., Stambler N., Malta E., Fuller C., Hilt D., Thurmond B., Nakanishi A. (1999). The ALSFRS-R: A Revised ALS Functional Rating Scale That Incorporates Assessments of Respiratory Function. BDNF ALS Study Group (Phase III). J. Neurol. Sci..

[26] Nagana Gowda G.A., Gowda Y.N., Raftery D. (2015). Expanding the Limits of Human Blood Metabolite Quantitation Using NMR Spectroscopy. Anal. Chem..

[27] Wishart D.S., Feunang Y.D., Marcu A., Guo A.C., Liang K., Vázquez-Fresno R., Sajed T., Johnson D., Li C., Karu N. (2018). HMDB 4.0: The Human Metabolome Database for 2018. Nucleic Acids Res.

[28] Pang Z., Chong J., Zhou G., de Lima Morais D.A., Chang L., Barrette M., Gauthier C., Jacques P.-É., Li S., Xia J. (2021). MetaboAnalyst 5.0: Narrowing the Gap between Raw Spectra and Functional Insights. Nucleic Acids Res..

[29] Liu M., Tang H., Nicholson J.K., Lindon J.C. (2002). Use of 1H NMR-Determined Diffusion Coefficients to Characterize Lipoprotein Fractions in Human Blood Plasma. Magn. Reson. Chem..

[30] Hendriks M.M.W.B., van Eeuwijk F.A., Jellema R.H., Westerhuis J.A., Reijmers T.H., Hoefsloot H.C.J., Smilde A.K. (2011). Data-Processing Strategies for Metabolomics Studies. TrAC Trends Anal. Chem..

[31] Kuhn M., Johnson K. (2013). Applied Predictive Modeling.

[32] Chicco D., Tötsch N., Jurman G. (2021). The Matthews Correlation Coefficient (MCC) Is More Reliable than Balanced Accuracy, Bookmaker Informedness, and Markedness in Two-Class Confusion Matrix Evaluation. BioData Min..

[33] Sinaki M., Mulder D.W. (1986). Amyotrophic Lateral Sclerosis: Relationship between Serum Creatine Kinase Level and Patient Survival. Arch. Phys. Med. Rehabil..

[34] Lawton K.A., Cudkowicz M.E., Brown M.V., Alexander D., Caffrey R., Wulff J.E., Bowser R., Lawson R., Jaffa M., Milburn M.V. (2012). Biochemical Alterations Associated with ALS. Amyotroph. Lateral Scler..

[35] Lawton K.A., Brown M.V., Alexander D., Li Z., Wulff J.E., Lawson R., Jaffa M., Milburn M.V., Ryals J.A., Bowser R. (2014). Plasma Metabolomic Biomarker Panel to Distinguish Patients with Amyotrophic Lateral Sclerosis from Disease Mimics. Amyotroph. Lateral Scler. Front. Degener..

[36] Rosenfeld J., King R.M., Jackson C.E., Bedlack R.S., Barohn R.J., Dick A., Phillips L.H., Chapin J., Gelinas D.F., Lou J.-S. (2008). Creatine Monohydrate in ALS: Effects on Strength, Fatigue, Respiratory Status and ALSFRS. Amyotroph. Lateral Scler..

[37] Shefner J.M., Cudkowicz M.E., Schoenfeld D., Conrad T., Taft J., Chilton M., Urbinelli L., Qureshi M., Zhang H., Pestronk A. (2004). A Clinical Trial of Creatine in ALS. Neurology.

[38] Ellis A.C., Rosenfeld J. (2004). The Role of Creatine in the Management of Amyotrophic Lateral Sclerosis and Other Neurodegenerative Disorders. CNS Drugs.

[39] Corbett A.J., Griggs R.C., Moxley R.T. (1982). Skeletal Muscle Catabolism in Amyotrophic Lateral Sclerosis and Chronic Spinal Muscular Atrophy. Neurology.

[40] Hayamizu K. (2017). Amino Acids and Energy Metabolism.

[41] Mann G., Mora S., Madu G., Adegoke O.A.J. (2021). Branched-Chain Amino Acids: Catabolism in Skeletal Muscle and Implications for Muscle and Whole-Body Metabolism. Front. Physiol..

[42] Tumani H., Shen G., Peter J.B., Brück W. (1999). Glutamine Synthetase in Cerebrospinal Fluid, Serum, and Brain: A Diagnostic Marker for Alzheimer Disease?. Arch. Neurol..

[43] Ouyang H., Gao X., Zhang J. (2022). Impaired Expression of BCAT1 Relates to Muscle Atrophy of Mouse Model of Sarcopenia. BMC Musculoskelet. Disord..

[44] Holecek M. (2001). Effect of Starvation on Branched-Chain Alpha-Keto Acid Dehydrogenase Activity in Rat Heart and Skeletal Muscle. Physiol. Res..

[45] The Italian ALS Study Group (1993). Branched-Chain Amino Acids and Amyotrophic Lateral Sclerosis. Neurology.

[46] Tandan R., Bromberg M.B., Forshew D., Fries T.J., Badger G.J., Carpenter J., Krusinski P.B., Betts E.F., Arciero K., Nau K. (1996). A Controlled Trial of Amino Acid Therapy in Amyotrophic Lateral Sclerosis. Neurology.

[47] Testa D., Caraceni T., Fetoni V. (1989). Branched-Chain Amino Acids in the Treatment of Amyotrophic Lateral Sclerosis. J. Neurol..

[48] Yoo H.-S., Shanmugalingam U., Smith P.D. (2022). Potential Roles of Branched-Chain Amino Acids in Neurodegeneration. Nutrition.

[49] De Felice A., Confaloni A., Crestini A., De Simone R., Malchiodi-Albedi F., Martire A., Matteucci A., Minghetti L., Popoli P., Venerosi A. (2015). Branched Chain Amino Acids in Experimental Models of Amyotrophic Lateral Sclerosis. Branched Chain Amino Acids in Clinical Nutrition.

[50] Conn A.R., Steele R.D. (1982). Transport of Alpha-Keto Analogues of Amino Acids across Blood-Brain Barrier in Rats. Am. J. Physiol. -Endocrinol. Metab..

[51] Hui S., Ghergurovich J.M., Morscher R.J., Jang C., Teng X., Lu W., Esparza L.A., Reya T., Zhan L., Guo J.Y. (2017). Glucose Feeds the TCA Cycle via Circulating Lactate. Nature.

[52] Xia J., Broadhurst D.I., Wilson M., Wishart D.S. (2013). Translational Biomarker Discovery in Clinical Metabolomics: An Introductory Tutorial. Metabolomics.

[53] Chang K.-H., Lin C.-N., Chen C.-M., Lyu R.-K., Chu C.-C., Liao M.-F., Huang C.-C., Chang H.-S., Ro L.-S., Kuo H.-C. (2021). Altered Metabolic Profiles of the Plasma of Patients with Amyotrophic Lateral Sclerosis. Biomedicines.

[54] Jia R., Chen Q., Zhou Q., Zhang R., Jin J., Hu F., Liu X., Qin X., Kang L., Zhao S. (2021). Characteristics of Serum Metabolites in Sporadic Amyotrophic Lateral Sclerosis Patients Based on Gas Chromatography-Mass Spectrometry. Sci. Rep..

[55] López-López Á., López-Gonzálvez Á., Barker-Tejeda T.C., Barbas C. (2018). A Review of Validated Biomarkers Obtained through Metabolomics. Expert Rev. Mol. Diagn..

